# Supplementary material for: The interactome of intact mitochondria by cross-linking mass spectrometry provides evidence for coexisting respiratory supercomplexes
Source: Mol Cell Proteomics. 2017 Dec 8;17(2):216–32. doi: 10.1074/mcp.RA117.000470 (PMC5795388; doi:10.1074/mcp.RA117.000470)
Supplement: Supplemental Data [file supp_RA117.000470_133922_0_supp_23978_fzffwf.zip › spectra_annotation/mito_DR_spectra_annotation/138-1-2-1-4-1.pdf]

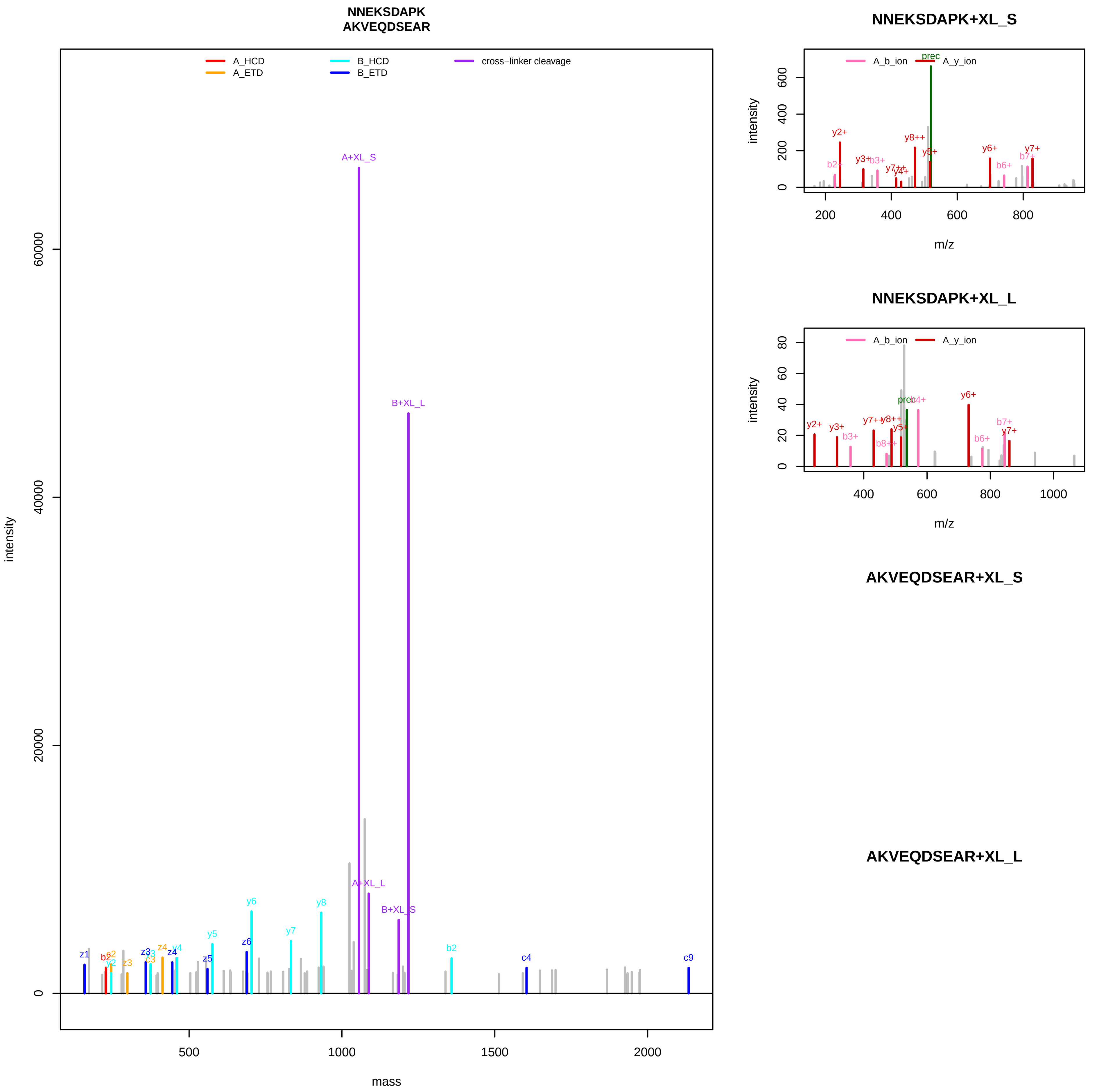
NNEKSDAPK
AKVEQDSEAR
A_HCD
A_ETD
B_HCD
B_ETD
cross−linker cleavage
A+XL_S
B+XL_L
A+XL_L
B+XL_S
z1
b2
c2
y2
z3
c3
z4
y4
y5
z5
z6
y6
y7
y8
b2
c4
c9
intensity
mass
0
20000
40000
60000
500
1000
1500
2000
NNEKSDAPK+XL_S
A_b_ion
A_y_ion
prec
y2+
b2+
y3+
b3+
y8++
y5+
y6+
b6+
y7+
b7+
intensity
m/z
NNEKSDAPK+XL_L
A_b_ion
A_y_ion
prec
c4+
y2+
y3+
b3+
y7++
y8++
b8++
y5+
y6+
b6+
b7+
y7+
intensity
m/z
AKVEQDSEAR+XL_S
AKVEQDSEAR+XL_L
